# Novel Polymorphic Multilocus Microsatellite Markers to Distinguish *Candida tropicalis* Isolates

**DOI:** 10.1371/journal.pone.0166156

**Published:** 2016-11-07

**Authors:** Xin Fan, Meng Xiao, Ping Liu, Sharon Chen, Fanrong Kong, He Wang, Li Zhang, Xin Hou, Ying-Chun Xu

**Affiliations:** 1 Department of Clinical Laboratory, Peking Union Medical College Hospital, Chinese Academy of Medical Sciences, Beijing, China; 2 Graduate School, Peking Union Medical College, Chinese Academy of Medical Sciences, Beijing, China; 3 Department of Clinical Laboratory, Tianjin First Centre Hospital, Tianjin, China; 4 Centre for Infectious Diseases and Microbiology Laboratory Services, Westmead Hospital, Darcy Road, Westmead, New South Wales, Australia; Leibniz Institute for Natural Products Research and Infection Biology- Hans Knoell Institute, GERMANY

## Abstract

*Candida tropicalis* is an important pathogen. Here we developed and evaluated a polymorphic multilocus microsatellite scheme employing novel genetic markers for genotyping of *C*. *tropicalis*. Using 10 isolates from 10 unique (separate) patients to screen over 4000 tandem repeats from the *C*. *tropicalis* genome (strain MYA-3404), six new candidate microsatellite loci (ctm1, ctm3, ctm8, ctm18, ctm24 and ctm26) were selected according to amplification success, observed polymorphisms and stability of flanking regions by preliminary testing. Two known microsatellite loci CT14 and URA3 were also studied. The 6-locus scheme was then tested against a set of 82 different isolates from 32 patients. Microsatellite genotypes of isolates from the same patient (two to five isolates per patient) were identical. The six loci produced eight to 17 allele types and identified 11 to 24 genotypes amongst 32 patients’ isolates, achieving a discriminatory power (DP) of 0.76 to 0.97 (versus 0.78 for both CT14 and URA3 loci, respectively). Testing of a combination of only three loci, ctm1, ctm3 and ctm24, also achieved maximum typing efficiency (DP = 0.99, 29 genotypes). The microsatellite typing scheme had good correlation compared with pulsed-field gel electrophoresis, although was slightly less discriminatory. The new six-locus microsatellite typing scheme is a potentially valuable tool for genotyping and investigating microevolution of *C*. *tropicalis*.

## Introduction

The incidence of nosocomial infections caused by *Candida* species has increased significantly over recent decades, with invasive candidiasis, which includes bloodstream infections with *Candida* spp., associated with high mortality, and excess hospital costs [1–4]. Although *Candida albicans* remains the most common cause of invasive candidiasis, non-*albicans Candida* spp. are increasingly recognized [2, 5, 6].

*C*. *tropicalis* is an important non-*albicans Candida* pathogen particularly in patients with leukaemia and cancer [7]; however the frequency of *C*. *tropicalis* infections varies in different geographic regions [5, 7, 8]. The proportional frequency is particularly high in China where this species caused 15.1% of all invasive candidiasis in a multicentre study [5] and in India accounted for 41.6% of candidemia cases in an intensive care unit-based survey [9]. *C*. *tropicalis* also has a propensity to be associated with nosocomial outbreaks [10–12]. Furthermore, in some regions, increasing resistance to azoles has been reported in *C*. *tropicalis* isolates [13, 14]. Molecular epidemiological studies are required to understand the genetic relatedness of *C*. *tropicalis* isolates, especially in the transmission of disease, as well as to establish effective surveillance.

Several molecular approaches, e.g. restriction fragment length polymorphism (RFLP) [12], randomly amplified polymorphic DNA (RAPD) [15], pulse-field electrophoresis (PFGE) [16, 17] and multilocus sequence typing (MLST) [18–20] have been employed for the genotyping of *C*. *tropicalis*. As an alternative, analysis of microsatellite loci offers potential as a typing technique because it has good discriminatory power, is highly reproducible and easy-to-perform, and has already been successfully applied to discriminate among strains of *C*. *albicans* [21] and *C*. *parapsilosis* [22]. The aim of the present study was to identify new polymorphic microsatellite loci as genetic markers of *C*. *tropicalis* and to evaluate their applicability for genotyping, extending the set of markers with clinical utility.

## Methods

### Ethics

The Human Research Ethics Committee of Peking Union Medical College Hospital approved this study and waived the need for consent.

### Isolates and DNA extraction

**Fig 1:**
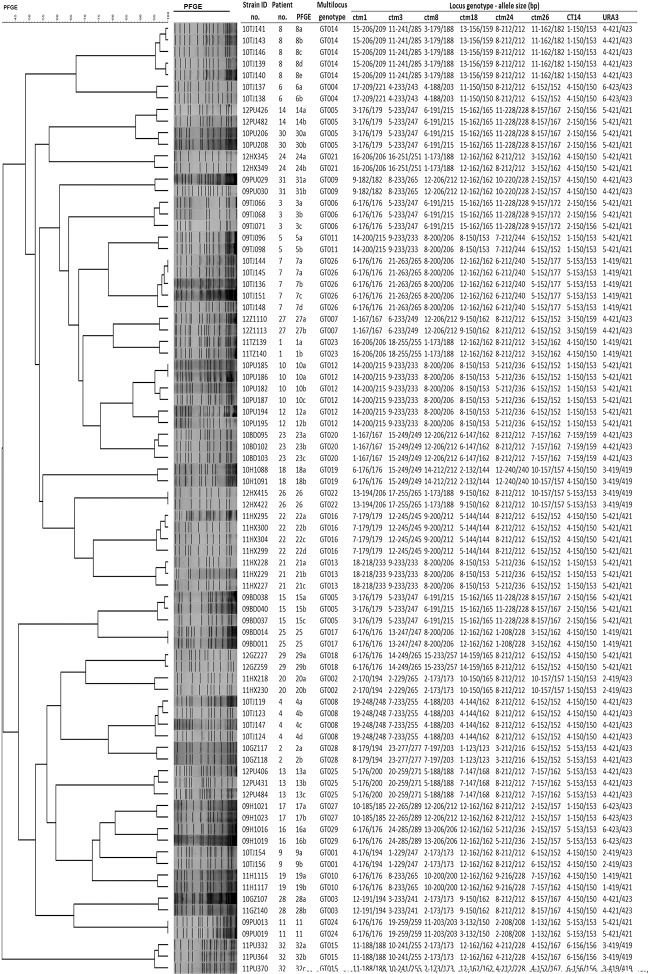
Unweighted pair group method using arithmetic average (UPGMA) dendrogram draw by pulsed-field gel electrophoresis (PFGE) typing results of 82 *C*. *tropicalis* isolates from 32 patient, genotype and allele sizes of eight microsatellite loci being evaluated, and multilocus microsatellite genotype of each isolate.

In this study, two sets of *C*. *tropicalis* clinical isolates were used: i) 10 unique isolates collected from 10 different patients in 10 different hospitals in 2010, for the development and preliminary evaluation of microsatellite typing scheme using selected loci, and reproducibility testing of the method; and ii) 82 *C*. *tropicalis* isolates from 32 patients with invasive candidiasis (two to five isolates per patient, see Fig 1) selected from the China Hospital Invasive Fungal Surveillance Net (CHIF-NET) programme. They were collected from eight different hospitals in seven provinces in mainland China from August 2009 to July 2012. Of the 82 isolates, 33 were from blood culture specimens, 26 from ascitic fluid, seven from pleural fluid, six from bile, four from venous catheter and two of each from pus, cerebrospinal fluid and bronchoalveolar lavage fluid. All isolates were identified to species level at the Department of Clinical Laboratory, Peking Union Medical College Hospital by matrix-assisted laser desorption ionization-time of flight mass spectrometry (MALDI-TOF MS) using the Bruker Biotyper system (Database DB 5627, v.3.1 was applied)(Bruker Daltoniks, Bemen, Germany), and stored at −80°C until being used.

Three reference strains–*C*. *albicans* ATCC 90028, *C*. *parapsilosis* sensu stricto ATCC 22019, *Candida krusei* ATCC 6258—and one clinical isolate each of *Candida glabrata* sensu stricto, *Candida guilliermondii* and *Candida metapsilosis* and *Cryptococcus neoformans* obtained from CHIF-NET programme were also included to evaluate the specificity of the microsatellite typing scheme developed in this study.

Prior to DNA extraction, isolates were subcultured onto Sabourauds dextrose agar for 24 h at 35°C. DNA extraction was performed using a glass bead and heating-assisted QIAamp DNA mini kit (Qiagen, Hilden, Germany) method as described by Metwally *et al*. [23] and stored at −20°C before use.

### Screening of potential microsatellite loci

The genome of *C*. *tropicalis* strain MYA-3404 available in NCBI genome database (accession number AAFN00000000.2) was used as the reference sequence, and potential microsatellite loci were screened using Tandem Repeat Finder software [24]. Twenty-six loci were selected (S1 Table), and 26 pairs of non-labelled primers were designed on the upstream and downstream non-variable flanking regions of each locus, for locus-specific amplification with Primer5 software (Plymouth Marine Laboratory, Plymouth, UK).

Ten *C*. *tropicalis* clinical isolates were used for the preliminary evaluation of the 26 selected microsatellite loci by singleplex PCR. For amplification, each PCR mix contained 12.5 μl of 2× EasyTaq PCR SuperMix (TransGen Biotech, Beijing, China), 2 μl of DNA template, 0.5 μM of forward and reverse primers, and molecular biology grade water (TransGen Biotech) was added to make a total volume of 25 μl. PCR was performed as follows: initial denaturation at 95°C for 10 min, followed by 30 cycles of denaturation at 94°C for 30 sec, annealing at 55°C for 30 sec, extension at 72°C for 45 sec, with a final extension at 72°C for 10 min. PCR products were analysed on a 1.5% agarose gel by electrophoresis. DNA sequencing was carried out for each locus by using the amplification primers from both directions. Polymorphic sequences of heterozygous isolates at each heterozygous loci were read and interpreted manually. Then the presence of expected tandem repeat motifs and stability of DNA sequences in flanking regions were confirmed (S1 Fig).

### Microsatellite analysis

After removing those microsatellite loci yielding amplification failure or unstable flanking regions in the preliminary experiments, six loci, namely ctm1, ctm3, ctm8, ctm18, ctm24 and ctm26, were selected for further study (Table 1 and S1 Table). In addition, two additional microsatellite loci recommended by Desnos-Ollivier *et al*., CT14 and URA3 (Table 1) [25], were also utilised for comparative evaluation, hence the total number of microsatellite loci evaluated was eight. For microsatellite analysis, singleplex PCR for each selected locus were performed as in the preliminary evaluation stage (see above), with forward primers of each loci being modified with 5’-end fluorescent (6-carboxyfluorescein [FAM], 6-carboxyhexafluorescein [HEX] or 6-carboxytetramethylrhodamine [TAMRA]) labelled (Table 1).

**Table 1:** Primers for selected microsatellite loci being evaluated in this study.

**Locus**	Motif	Forward/reverse primer sequences	5' end-label of primer	Reference
ctm1^a^	(AGA)_12_	TGGAAGTTACATAATGGTGATAAGTTC	FAM	This study
		/GATATGCTTTATGCCTGGAATAG	None	
ctm3^a^	(AG)_26_	ACTCACCCACTCACACAAAAC	HEX	This study
		/CGTTATAAGTAAATCTTGATGATTCG	None	
ctm8^a^	(TCA)_19_	TCAACATGACTATCATCATCTTCAG	FAM	This study
		/GATGATGACAATGACGTTGATATCTC	None	
ctm18^a^	(TTC)_19_	CCAATCCCTTATTCAACAATTAATATAC	HEX	This study
		/GCAGCTTTACCAATAATTGACATT	None	
ctm24^a^	(TTTA)_12_	CACATTAATATTACCTCGAACGTG	TAMRA	This study
		/CTAAAGGCGGGTATAGTTTATTGG	None	
ctm26^a^	(TATTT)_11_	CATTTCAATACCTGATAATTCTCCTC	FAM	This study
		/CTTAGACAAGGCTCTACAGCACT	None	
CT14	(TGA)_7_	GTAAATCTTGTATACCGTGGA	FAM	Desnos-Ollivier M *et al*. [25]
		/TAGCCCATTTTCTAGTTTTGC	None	
URA3	(CA)_6_	ATTGGATAGTCCCTCTAAACTCACTACTA	HEX	Desnos-Ollivier M *et al*. [25]
		/AGCATTAGTTATATCACTCCACGATGAA	None	

Abbreviations: FAM, 6-carboxyfluorescein; HEX, 6-carboxyhexafluorescein; TAMRA, 6-carboxytetramethylrhodamine.

^a^See detail genetic information of these loci in S1 Table.

**Fig 2:**
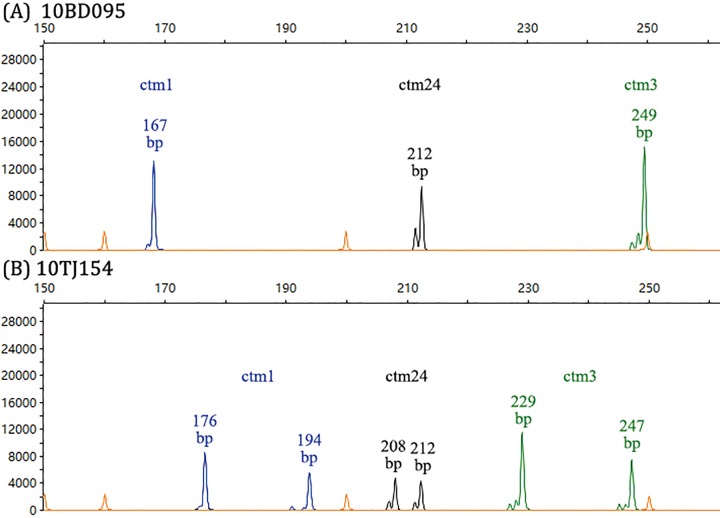
Microsatellite typing results with markers ctm1 (FAM-labelled, shown in blue), ctm3 (HEX-labelled, shown in green) and ctm24 (TAMRA-labelled, shown in black). The GeneScan™ 500 LIZ (Applied Biosystems) size standard was shown in orange. (A) Strain 10BD095, which was homozygous at all three tested loci. (B) Strain 10TJ154, which was heterozygous at all three loci.

The PCR product was then diluted 1:10 in water. Fragment separation was performed on an ABI 3730 sequencer (Applied Biosystems, Carlsbad, USA) with a 50 cm POP 7 gel. Sample injection was at 1.6 kV over 15 seconds, with a total running time of 6200 seconds. A GeneScan™ 500 LIZ (Applied Biosystems) size standard was used as internal marker. The results were analyzed by GeneMarker software (Version2.2.0, SoftGenetics, State College, PA, USA). Isolates with two PCR amplicons detected were considered as heterozygous, while strains presenting a single amplification product were considered homozygous (Fig 2). *C*. *tropicalis* strain 10BD095 (Fig 2) were included in each run as an internal quality control of experiment.

The discriminatory power (DP) for each marker was calculated using the Simpson index as follows: DP = 1-$\frac{1}{N(N-1)}\sum_{j=1}^{S}nj(nj-1)$, in which *N* is the number of strains, *S* is the total number of different genotypes, and *nj* is the number of strains of genotype *j* [26].

### Reproducibility and species specificity

The 10 clinical isolates used in preliminary evaluation of the method were tested in technical triplicates, which were prepared by blinded technicians not being involved in previous microsatellite tests.

In addition, seven strains of seven non-*C*. *tropicalis* yeast species (see above) were used to test the inter-species specificity of the microsatellite loci selected. Amplification of strains’ internal transcribed spacer (ITS) region, as described previously [5], were carried out as positive controls to check DNA quality from these strains.

### PFGE

To compare the discriminatory power of microsatellite typing with PFGE, the latter was carried out on all 82 isolates as described previously [27] using the restriction enzyme BssHII. Assessment of PFGE types of isolates were performed using BioNumerics software (version 7.5, Applied Maths, Kortrijk, Belgium) by using Dice coefficient, and dendrogram analysis performed with the unweighted pair group method using arithmetic average (UPGMA). Isolates were considered different types when the band similarity value was less than 95% [16], and assigned to different subtypes when ≥1 band differences were observed.

### Optimization of microsatellite analysis system

To reduce the complexity of microsatellite analysis whilst maintaining its high DP, different combinations of microsatellite loci were further assessed, of which the combination of loci, ctm1, ctm3 and ctm24, was considered optimal and will be the loci employed for future use in our laboratory (see Results). In addition, the feasibility of amplifying these three loci simultaneously by a triplex PCR using a mixture of the same primer pairs designed for singleplex PCRs was evaluated.

## Results

### Screening and selection of potential microsatellite loci

A total of 4288 tandem repeats, whose repeated motif was >1 bp, were archived from genome of *C*. *tropicalis* strain MYA-3404 (accession number AAFN00000000.2). The primary selection criteria for potential microsatellite loci used in the present study were that the: i) repeated motif be between 2 to 6 bp, ii) length of repeat sequences be between 50 to 100 bp, and iii) loci must be located outside of known coding regions, which have higher probability of showing greater genetic variability. On application of these criteria, 26 loci were initially selected (S1 Table).

On study of the 10 isolates used for the preliminary evaluation, 16 of the 26 candidate loci produced unsuccessful amplification and were rejected. In addition, four loci were observed to have unstable flanking regions by DNA sequencing (e.g. locus ctm16, S1 Fig) and these were no further evaluated. Targeting of the remaining six loci, ctm1, ctm3, ctm8, ctm18, ctm24 and ctm26, resulted in satisfactory PCR amplification, observed nucleotide polymorphisms and flanking region stability (S1 Fig), as did the loci CT14 and URA3 [25]. These eight loci were then further studied for their utility as genotyping markers (Table 1 and S1 Table and see below).

### Microsatellite analysis

The typing capacity of the eight selected microsatellite loci were evaluated using a different set of 82 *C*. *tropicalis* isolates from 32 patients. As *C*. *tropicalis* is a diploid species, one or two PCR amplicons were obtained for each locus (Fig 2), and each amplified fragment was assigned to an allele. Of note, all isolates from the same patient revealed same microsatellite genotype, implicating infection from a single infection episode (Fig 1). Therefore, only one isolate per patient (32 unique isolates from 32 patients) were used to calculating the discriminatory power of different microsatellite loci. For the eight loci, a number of 3–17 alleles were obtained, while 34.4% to 71.9% of the 32 patients’ isolates were heterozygous (Table 2 and Fig 1). The eight loci revealed different degrees of polymorphisms. Locus ctm3, which produced 24 genotypes, had the highest DP (DP = 0.97), followed by locus ctm1 (19 genotypes, DP = 0.93) and ctm8 (15 genotypes, DP = 0.92) (Table 2). All six loci identified in this study presented superior typing efficiency to loci CT14 (seven genotypes, DP = 0.78) and URA3 (six genotypes, DP = 0.78) (Table 2) [25].

**Table 2:** Characteristics of the microsatellite loci selected.

Characteristics	ctm1	ctm3	ctm8	ctm18	ctm24	ctm26	CT14	URA3	Eight loci	ctm1+ctm3+ctm24
No. alleles	17	16	12	11	8	8	4	3	N/A	N/A
Allele size range (bp)	167-	229-	173-	123-	208-	132-	150-	419-	N/A	N/A
	248	289	257	168	244	182	159	423		
Repeat number range	7–34	10–40	8–36	6–21	5–14	2–12	6–9	5–7	N/A	N/A
No. genotypes	19	24	15	15	12	11	7	6	29	29
Discriminator power	0.93	0.97	0.92	0.88	0.76	0.85	0.78	0.78	0.99	0.99
Allele frequency	0.016-	0.016-	0.016-	0.016-	0.016-	0.016-	0.047-	0.188-	N/A	N/A
	0.328	0.281	0.188	0.453	0.594	0.469	0.563	0.563		
Genotype frequency	0.031-	0.031-	0.031-	0.031-	0.031-	0.031-	0.031-	0.063-	0.031-	0.031-
	0.250	0.125	0.188	0.313	0.469	0.344	0.375	0.375	0.094	0.094
%Heterozygosity	46.9	62.5	71.9	59.4	34.4	56.3	37.5	43.8	N/A	N/A

Abbreviation: N/A, not applicable.

**Fig 3:**
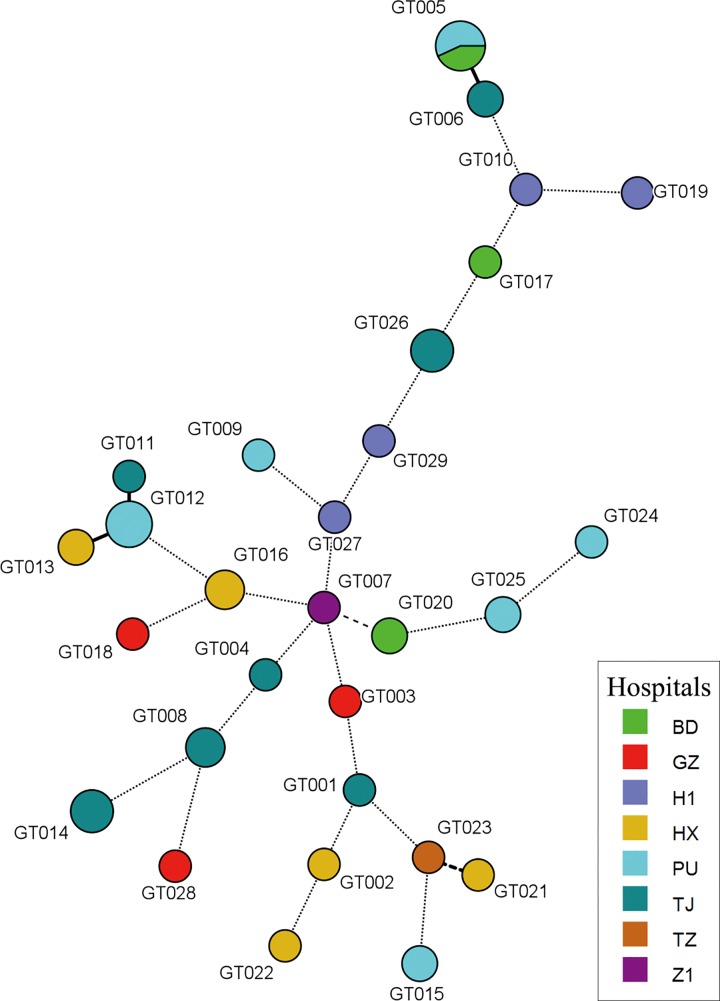
Minimum spanning tree analysis based on three-locus (ctm1, ctm3 and ctm24) microsatellite genotypes of isolates and hospital where the isolates were collected. Each circle corresponds to a microsatellite genotype, and different colour represented different hospitals (see hospital full names in Acknowledgment section).

The combination of using eight microsatellite loci revealed 29 genotypes among 32 patients’ isolates, and the overall DP was 0.99 (Table 2). Amongst different patients, patient no. 14 (with two isolates) and patient no. 30 (two isolates) from hospital PU and patient no.15 (three isolates) from hospital BD carried isolates with an identical microsatellite genotype–GT005 (Figs 1 and 2), and this was the only instance where an identical microsatellite genotype was shared by isolates from patients in different hospitals (Fig 3). In addition, patient no. 10 (with four isolates) and patient no. 12 (two isolates), both of whom were from hospital PU, carried isolates with the same microsatellite genotype GT012 (Fig 1). For the remaining 27 patients, each patient carried isolates with a unique microsatellite genotype (Fig 1). No clustering of genetically related *C*. *tropicalis* isolates in a specific hospital was observed (Fig 3).

### Species-specificity and reproducibility

When PCRs were carried out using primer pairs and conditions designed for *C*. *tropicalis* microsatellite typing against other common *Candida* species including *C*. *albicans*, *C*. *parapsilosis* sensu stricto, *C*. *krusei*, *C*. *glabrata*, *C*. *guilliermondii*, *C*. *metapsilosis* and *Cryptococcus neoformans*, no amplicon was observed. Hence, the microsatellite markers were *C*. *tropicalis* species-specific.

On testing 10 isolates in triplicate, the maximum inter-run size-calling differences for the same locus of the same strain were 0.2 bp. The microsatellite genotypes for the same strain were identical between different runs.

### Comparison of microsatellite typing with PFGE

PFGE analysis revealed 32 PFGE types and 77 subtypes amongst the 82 *C*. *tropicalis* isolates. All isolates from the same patient were of the same PFGE type (band similarity value ≥ 95%), whilst isolates from different patients were of different PFGE types (Fig 1). PFGE also revealed no clustering of genetically related *C*. *tropicalis* isolates in a specific hospital (Fig 1).

For isolates with identical microsatellite genotypes isolated from different patients, the six microsatellite GT012 isolates from two patients in hospital PU also clustered together with >85% band similarity by PFGE. However, of the above-mentioned seven microsatellite GT005 isolates from three patients (patients no. 14, no. 15 and no. 30) in two hospitals, four isolates from hospital PU (strains 12PU426 and 12PU482 from patient no.14, and strains 10PU206 and 10PU208 from patient no. 30) were clustered together by PFGE with band similarity value of >85%, but were clearly distinguished from the three isolates from hospital BD (strains 09BD037, 09BD038 and 09BD040, patient no. 15) (Fig 1).

### Optimization of microsatellite analysis system

To maintain the DP of microsatellite typing, attempts were made to reduce the number of loci being used. Loci ctm1 and ctm3 were selected initially because these presented the highest DP values. The ctm1-ctm3 combination was able to assign 27 of 29 locus microsatellite genotypes except for GT011 and GT012 (Fig 1). On addition of locus ctm24 to the analysis, the three-locus (ctm1-ctm3-ctm24) combination achieved the same DP (= 0.99) as the 8-locus typing scheme and was the only three-locus combination to do so (Table 2).

Genotyping results of the ctm1-ctm3-ctm24 triplex PCR scheme, shown that results were identical to those obtained by the three singleplex PCRs-based microsatellite genotyping results. The maximum size-calling difference for the same loci allele of the same strain between singleplex and triplex PCR schemes was 0.2 bp, and the average size-calling difference was 0.05 bp.

## Discussion

As one of the most common non-*albicans Candida* species causing invasive candidiasis, particularly in Asia [5, 6, 9], and with concern of fluconazole resistance in some regions [13, 14, 17], molecular typing is necessary for *C*. *tropicalis* to gain insight into disease transmission and to establish efficient surveillance. In this study we have developed a novel microsatellite-based genotyping scheme based on the use of six novel markers with the use of at least three loci in combination providing high discriminatory power for distinguishing between *C*. *tropicalis* strains. Specifically, the microsatellite typing method developed had good reproducibility and species-specificity, and enabled the designation of 29 microsatellite types amongst the 32 patients’ isolates challenged against the typing method. The six markers selected identified 8 to 17 alleles and 11 to 24 genotypes, of which the locus ctm3 had the highest typing efficiency (DP = 0.97).

Although microsatellite-based genotyping typing has been reported by others, the results in the present study demonstrate a number of differences highlighting the need to continue to search for additional, more specific, makers of *C*. *tropicalis*. Previously, Desnos-Ollivier *et al*. used a double-locus (CT14 and URA3) microsatellite scheme to genotype *C*. *tropicalis* [25]. As evaluated in the present study, each of CT14 and URA3 loci produced results that were less discriminatory compared with the six new loci identified in the present study (DP = 0.78 both for CT14 and URA3, respectively, versus 0.76 to 0.97 for the six new loci). Another 6-locus microsatellite typing scheme employing Ctrm1, Ctrm10, Ctrm12, Ctrm21, Ctrm24 and Ctrm28, was recently proposed by Wu *et al*.[15]. Although we did not adopt their typing scheme, we found that one locus, namely, Ctrm1 in that study and the locus ctm1 used in our study, were the same (S1 Table). In Wu *et al*.’s study, the Ctrm1/ctm1 locus exhibited highest DP value (DP = 0.95, versus 0.70–0.91 of other five loci) [15], whilst it was the only second most discriminatory locus in ours (DP = 0.93). Therefore, it is reasonable to conclude that the typing efficiency of microsatellite assay developed here is more discriminatory than that of Desnos-Ollivier *et al*., and, at least, as discriminatory as that of Wu *et al*.

To date, of molecular assays that have been applied for genotyping of *C*. *tropicalis*, PFGE has proved to be a high discriminatory typing method, including the investigation of outbreaks of infection [11, 28]. However, PFGE is hindered by its handling complexity, long turn-around time and poor inter-laboratory comparability (Table 3). In this study, a good correlation between the genetic profiles of *C*. *tropicalis* isolates obtained by PFGE and by microsatellite methods were found, although microsatellite was slightly less discriminatory. Amongst the 82 isolates challenged against the microsatellite typing method and PFGE, all isolates with the same PFGE types had identical microsatellite types, and isolates with non-related microsatellite genotypes had different PFGE types. Only two patients in hospital PU and a patient in hospital BD had isolates sharing the same microsatellite genotype (GT005) but with different PFGE types (two genetic related PFGE types 14 and 30 from two patients in PU versus a divergent PFGE type 15 from the patient in BD). Therefore, as microsatellite typing is more practically feasible and PFGE provided higher typing resolutions, microsatellite typing could be recommended as a primary assay for typing of *C*. *tropicalis* in phylogenetic investigations, and PFGE would be a useful complementary tool to assist the interpretation of identical or closely-related microsatellite genotypes, especially for observing microevolutions or for isolates from a short period of time (such as outbreaks) [16].

**Table 3:** Features of the most frequently used typing methods for *C*. *tropicalis*.

Characters	PFGE	Microsatellite	MLST	RAPD
Discriminatory power	Highest	Very high	High	Low
Standardization	Poor	Good	Good	Poor
Inter-laboratory comparability	Poor	Good	Good	Poor
Experiment simplicity	Complicate	Simple	Moderate	Simple
Data analysis simplicity	Moderate	Simple	Complicate	Moderate
Turn-around time	Very long	Short	Moderate	Short
Running costs	Moderate	Low	High	Low
Proposed usage	Outbreak investigation	Outbreak investigation	Epidemiology study	Pathogen identification
	Microevolution detection	Epidemiology study	Evolution study	

Abbreviations: PFGE, pulsed-field gel electrophoresis; MLST, multilocus sequence typing; RAPD, randomly amplified polymorphic DNA.

There were other molecular assays that have been applied for genotyping of *C*. *tropicalis*. MLST, which is widely studied as a typing tool, produces results that correlate with those of PFGE [16, 17] but also has advantages in achieving interchangeable data for inter-laboratory comparisons and geographic population studies (Table 3) [16–20]. However, as *C*. *tropicalis* was a diploid eukaryotic species [18, 20], all sequencing results have to be checked manually to ensure heterozygosity, which increases the workload and likelihood of error. RAPD, is now considered by many to have suboptimal reproducibility and difficult to standardize (Table 3) [15]. In comparison, microsatellite analysis has the advantage of incorporating markers that evolve rapidly within the genome, offering good discriminatory power, reproducibility and portability, and is less costly [29, 30].

For the purpose of practicality, e.g. in studying a large scale of isolates in nationwide surveillance, we further simplified the microsatellite typing scheme used in the present study to incorporate only three loci, namely ctm1, ctm3 and ctm24, where in combination, produced consistent and excellent typing efficiency (DP = 0.99) versus using all loci (DP = 0.99). In addition, generation of DNA of sufficiently high quality for microsatellite typing was able to be achieved using a triplex PCR assay (rather than three individual singleplex PCR reactions), which further increase the efficiency of the whole procedure. However, when the primary goal is the general overview of the *C*. *tropicalis* genome or for microevolution studies, despite of the good DPs of the microsatellite loci identified herein, the distribution of different microsatellite markers in the genome should also be investigated and we are continuing to search for more microsatellite loci that could be of utility.

One limitation of the study was that, 10 of 26 loci being screened in preliminary experiment had amplification failures but were not further studied. These failures could be related to unexpected polymorphisms in the flanking regions where the primers were chosen, as unstable flanking regions upper- and down-stream of microsatellite repeat regions were observed (e.g. at locus ctm16) and in previous studies [15] by DNA sequencing. Another issue was that, it has been indicated that shorter repeat units may have higher proportions of technical artefacts called stutter peaks. In the present study, locus ctm3 were the only locus with dinucleotide repeats, and stutter peaks were observed; however, as the stutter peaks of ctm3 were insignificant (as the case shown in Fig 2), their presence unlikely influenced the interpretation of results and hence, the ctm3 locus was were still retained as a high discriminatory microsatellite marker.

## Conclusions

In conclusion, the new microsatellite typing system here has good potential as a tool for genotyping of *C*. *tropicalis*, which is simple, has good discriminatory power and reproducibility, and is less expensive than sequencing-based assays. In addition, the new typing system showed superior discriminatory power compared those used in previous *C*. *tropicalis* studies, and offers improved microsatellite markers. It has promising application in epidemiology and population evolution studies of *C*. *tropicalis*. Further evaluation of inter-laboratory comparability of the methods, as well as development of a standardized global interchangeable database will be of value.

## Supporting Information

S1 Fig Sequence alignment of different alleles of the ctm16 marker, showing the number of repeats of each allele (repeat motif ACTA, alignment positions 92–135 bp) and unstable flanking regions (alignment positions 29–44 bp). (DOCX)

S1 Table Genetic information for 26 potential microsatellite loci achieved from genome of *C*. *tropicalis* strain MYA-3404 (genome accession number AAFN00000000.2). (DOCX)
