# Supplementary material for: Novel Polymorphic Multilocus Microsatellite Markers to Distinguish Candida tropicalis Isolates
Source: PLoS One. 2016 Nov 7;11(11):e0166156. doi: 10.1371/journal.pone.0166156 (PMC5098789; doi:10.1371/journal.pone.0166156)
Supplement: S1 Fig — (DOCX) [file pone.0166156.s001.docx]

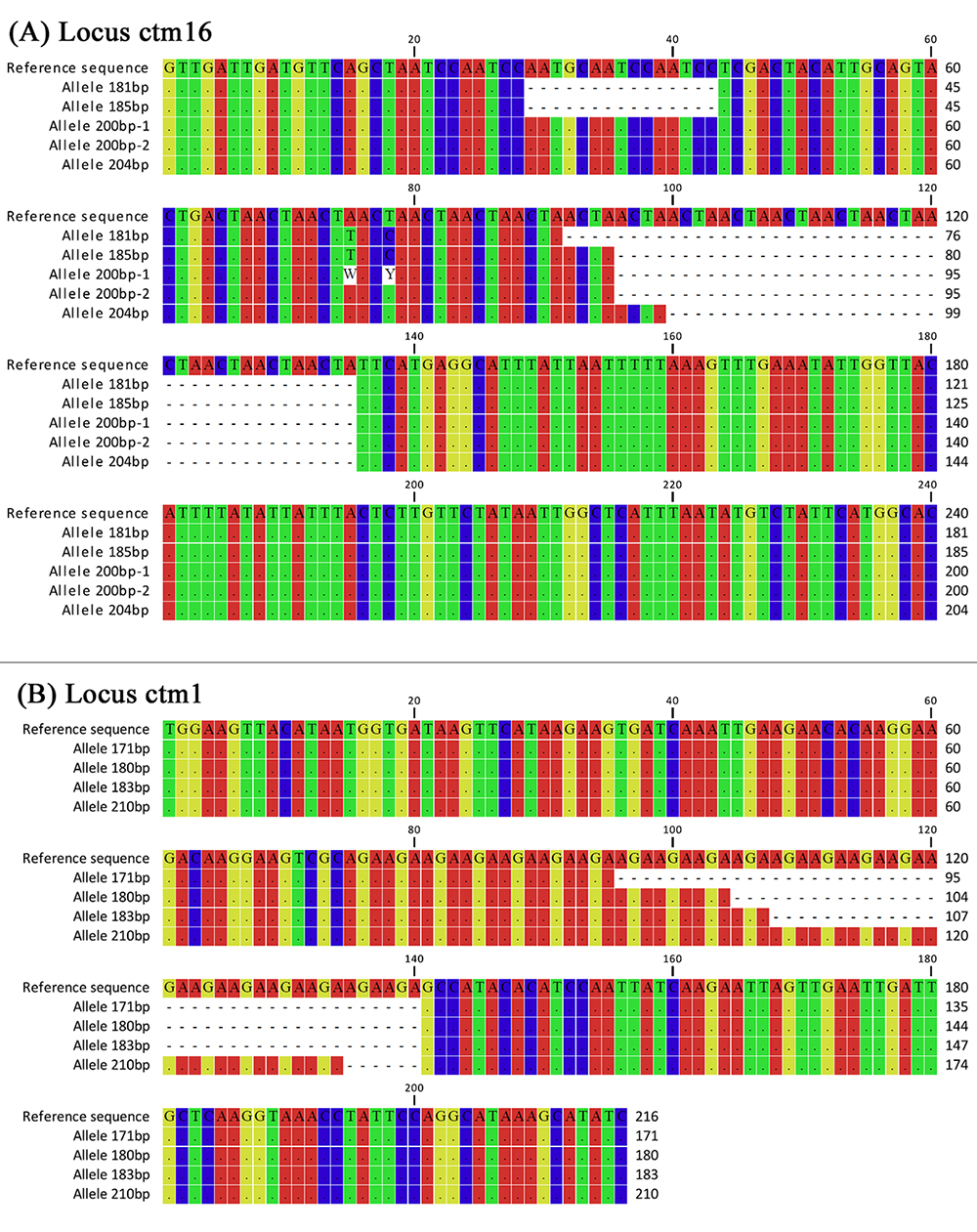


**S1 Figure. Sequence alignment for part of different alleles.** Reference sequence were from *C. tropicalis* genome strain MYA-3404. (A) Locus ctm16, showing the number of repeats of each allele (repeat motif ‘ACTA’, alignment positions 92-135 bp) and unstable flanking regions (alignment positions 29-44 bp). (B) Locus ctm1, showing the number of repeats (repeat motif ‘AGA’, alignment positions 96-140 bp) and stable flanking regions.
