# Supplementary material for: Novel Polymorphic Multilocus Microsatellite Markers to Distinguish Candida tropicalis Isolates
Source: PLoS One. 2016 Nov 7;11(11):e0166156. doi: 10.1371/journal.pone.0166156 (PMC5098789; doi:10.1371/journal.pone.0166156)
Supplement: S1 Table — (DOCX) [file pone.0166156.s002.docx]

**S1 Table. Genetic information for 26 potential microsatellite loci achieved from genome of *C. tropicalis* strain MYA-3404 (genome accession number AAFN00000000.2).**

| **Locus** | **Motif** | **Location** | **GenBank accession no.** | **Start position** | **End position** | **Copy number** |
| --- | --- | --- | --- | --- | --- | --- |
| ctm1 | AGA | Supercont3.1 | GG692395.1 | 31486 | 31551 | 22 |
| ctm2 | GAA | Supercont3.1 | GG692395.1 | 1168173 | 1168262 | 30 |
| ctm3 | AG | Supercont3.1 | GG692395.1 | 1276351 | 1276402 | 26 |
| ctm4 | CCTCTT | Supercont3.1 | GG692395.1 | 1805772 | 1805861 | 15 |
| ctm5 | GAA | Supercont3.1 | GG692395.1 | 1856927 | 1856977 | 17 |
| ctm6 | TTGCTG | Supercont3.1 | GG692395.1 | 2237179 | 2237226 | 8 |
| ctm7 | ATTCAT | Supercont3.1 | GG692395.1 | 2327641 | 2327700 | 10 |
| ctm8 | TCA | Supercont3.2 | GG692396.1 | 1339943 | 1339999 | 19 |
| ctm9 | TCATCT | Supercont3.2 | GG692396.1 | 1340405 | 1340452 | 8 |
| ctm10 | GA | Supercont3.2 | GG692396.1 | 1416533 | 1416584 | 26 |
| ctm11 | AAAGAG | Supercont3.2 | GG692396.1 | 1557953 | 1558000 | 8 |
| ctm12 | TCA | Supercont3.3 | GG692397.1 | 62649 | 62702 | 18 |
| ctm13 | GAAGCA | Supercont3.3 | GG692397.1 | 447361 | 447420 | 10 |
| ctm14 | AAT | Supercont3.3 | GG692397.1 | 456004 | 456060 | 19 |
| ctm15 | AC | Supercont3.3 | GG692397.1 | 954448 | 954525 | 39 |
| ctm16 | ACTA | Supercont3.3 | GG692397.1 | 975651 | 975722 | 18 |
| ctm17 | AAT | Supercont3.3 | GG692397.1 | 1976748 | 1976828 | 27 |
| ctm18 | TTC | Supercont3.4 | GG692398.1 | 1049797 | 1049853 | 19 |
| ctm19 | ACA | Supercont3.5 | GG692399.1 | 230130 | 230189 | 20 |
| ctm20 | TC | Supercont3.6 | GG692400.1 | 72462 | 72513 | 26 |
| ctm21 | AAG | Supercont3.6 | GG692400.1 | 386367 | 386435 | 23 |
| ctm22 | ATG | Supercont3.7 | GG692401.1 | 69764 | 69823 | 20 |
| ctm23 | CTA | Supercont3.7 | GG692401.1 | 116785 | 116841 | 19 |
| ctm24 | TTTA | Supercont3.7 | GG692401.1 | 712798 | 712845 | 12 |
| ctm25 | TCA | Supercont3.8 | GG692402.1 | 306802 | 306849 | 16 |
| ctm26 | TATTT | Supercont3.8 | GG692402.1 | 770658 | 770712 | 11 |
